# Improving engraftment in autologous hematopoietic stem cell transplantation: comparing pegfilgrastim with filgrastim in an outpatient setting

**DOI:** 10.1016/j.htct.2026.106436

**Published:** 2026-07-30

**Authors:** Cesar Homero Gutierrez-Aguirre, Fernando De la Garza-Salazar, David Gómez-Almaguer, José Carlos Jaime-Pérez, María del Consuelo Mancias-Guerra, Andrés Gómez-de León, Perla Colunga-Pedraza, Mariana Jazmín Moedano Mata, Verónica del Carmen Estrada Rosales, Olga Graciela Catú-Rodríguez

**Affiliations:** a Servicio de Hematología, Hospital Universitario “Dr. José Eleuterio González”, Universidad Autónoma de Nuevo León, Mexico; b Facultad de Medicina de la Universidad Autónoma de Nuevo León, Mexico

**Keywords:** Pegfilgrastim, Filgrastim, Febrile neutropenia, Autologous stem cell transplantation, Multiple myeloma

## Abstract

**Background:**

Febrile neutropenia, a frequent complication in patients undergoing autologous stem cell transplantation, increases morbidity and hospitalization costs.

**Objective:**

The aim of this study was to compare the efficacy of two formulations of pegylated filgrastim with filgrastim in patients who received autologous stem cell transplantation as outpatients for lymphoma or myeloma.

**Methods:**

Thirty patients were randomized to receive a single 6 mg dose of either reference or biosimilar pegfilgrastim on Day +1. A retrospective Control Group of fifty-three patients who received filgrastim was included.

**Main results:**

The median times to neutrophil engraftment were 10, 9 and 11 days for the innovator pegfilgrastim, biosimilar pegfilgrastim (p-value = 0.14), and filgrastim (p-value = 0.0001), respectively. The median times to platelet engraftment were 10 days for both of the pegfilgrastim groups and 12 days for the filgrastim group (p-value = 0.0001). Febrile neutropenia incidence was lower in the pegfilgrastim group (6.7%) than in the filgrastim group (24.5%; p-value = 0.04). Cost analysis showed higher costs for the innovator pegfilgrastim, with filgrastim having the lowest cost of the three formulations.

**Conclusion:**

The innovator and the biosimilar pegfilgrastim demonstrated similar efficacy in engraftment speed and febrile neutropenia incidence. Pegfilgrastim was associated with faster engraftment, a lower incidence of platelet transfusion, and a lower incidence of febrile neutropenia.

## Introduction

High-dose chemotherapy followed by autologous stem cell transplantation (ASCT) is the standard care provided to patients with relapsed/refractory lymphoma or with newly diagnosed multiple myeloma (MM) [1]. Although the best conditioning regimen for ASCT remains undefined, regimens such as BEAM (carmustine, etoposide, cytarabine, and melphalan) and high-dose melphalan (140–200 mg/m^2^) are the regimens of choice for lymphoma and myeloma, respectively. Importantly, outpatient transplant programs aim to avoid hospitalization to improve the quality of life of patients, the patient experience, and healthcare expenditure, reducing the total cost of ASCT by up to 50 % [2,3]. However, regardless of the conditioning regimen used and the follow-up model selected, febrile neutropenia (FN) is a common complication in the aplastic phase. During the post-conditioning period, neutropenic patients are at risk of infection, hospitalization, and the need for antibiotic therapy or blood transfusions.

Filgrastim, which is a recombinant granulocyte colony-stimulating factor (G-CSF), has traditionally been used to reduce the duration and severity of neutropenia during ASCT. After administering G-CSF at a dose of 10 mg/kg/day for 6–10 days, hematological recovery (neutrophil count >0.5 × 10^9^/L and platelet count >20 × 10^9^/L) is generally achieved between Day 12 and Day 14 (range: Day 10–25) post-transplant [4,5]. Pegfilgrastim is a long-acting form of G-CSF that was approved in 2002 and has since been widely used for the prophylaxis of chemotherapy-induced FN and, less frequently, in patients receiving ASCT. This study was designed to compare the efficacy of a single dose of either the original (innovator) pegylated filgrastim drug or a biosimilar pegylated filgrastim with the efficacy of daily filgrastim administration in patients who received ASCT as outpatients, with a particular focus on the time to engraftment.

## Methods

This was a prospective open-label, phase II, single-center study conducted in a public hospital in Monterrey, Mexico. It compared the effectiveness of three treatments for the prophylaxis of FN in patients receiving ASCT for lymphoma or myeloma as outpatients at the study center between March 2022 and November 2022. The treatment regimens consisted of: a single dose of innovator pegfilgrastim (Neulastim, Amgen, Mexico), a single dose of biosimilar pegfilgrastim (Linkix, Teva, Mexico), or daily treatment with biosimilar filgrastim.

## Patient population

Patients were eligible to participate in this study if they were between 18 and 75 years old; had a previous confirmed diagnosis of MM, non-Hodgkin lymphoma (NHL), or Hodgkin’s lymphoma (HL); and were in a first or consecutive remission after receiving an ASCT. Additional inclusion criteria were as follows: patients had an Eastern Cooperative Oncology Group (ECOG) performance status of <2, patients were eligible for ASCT, patients had not undergone chemotherapy as a mobilization regimen; and patients had normal liver function test results. The exclusion criteria comprised having one of the following conditions: fever; an active infection, including viral infections such as hepatitis A, B, or human immunodeficiency virus (HIV); or congestive heart failure with an ejection fraction of <40 %.

The study consisted of two arms: patients in Arm 1 received the innovator pegfilgrastim (Neulastim®, AMGEN, Mexico) for the prophylaxis of FN, whereas patients in Arm 2 received a biosimilar pegfilgrastim (Linkix®, TEVA, Mexico).

Additionally, a historical Control Group of 53 consecutively-treated patients who met the same inclusion criteria and received a biosimilar filgrastim (Biofilgran, Landsteiner Scientific, Mexico) for the prophylaxis of FN were included. Patients in this retrospective cohort received their transplant between January 2020 and December 2021, following the same outpatient follow-up protocols, antimicrobial prophylaxis, and hospitalization criteria established in the institutional standard operating procedure outlined in the unit's FACT (Foundation for the Accreditation of Cellular Therapy) accreditation.

## Conditioning regimen, stem cells, and infection prophylaxis

Patients with lymphoma received a conditioning regimen of bendamustine (180 mg/m^2^/day for two days) and melphalan (140 mg/m^2^/day for one day), or of etoposide (450 mg/m^2^/day for two days) and melphalan (140 mg/m^2^/day for one day). Patients with myeloma received melphalan (140 or 200 mg/m^2^/day for one day) (Table 1). The conditioning regimen to be used was chosen according to the availability of the medicines. Peripheral blood was the source of the hematopoietic stem cells in all patients. Hematopoietic stem cells were mobilized by the subcutaneous administration of filgrastim at 10 µg/kg/day for four consecutive days before being collected by apheresis on the fifth day of mobilization. Apheresis was performed as an outpatient procedure for all patients. The objective of this procedure was to obtain at least 2 × 10^6^/kg CD34^+^ hematopoietic stem cells. Infection prophylaxis for all patients consisted of oral levofloxacin (500 mg/day), acyclovir (400 mg/day), and voriconazole (200 mg/day). This regimen began on Day 0 (transplant day) and continued until absolute neutrophil count exceeded 500 cells/µL, at which point levofloxacin was replaced with trimethoprim-sulfamethoxazole.

**Table 1:** Characteristics of patients and conditioning schemes used.

	Characteristic	Arm 1 (Innovator pegfilgrastim)*n* = 15	Arm 2(Biosimilar pegfilgrastim)*n* = 15	Total*n* = 30	p-value
**Age in years, median, (range)**		47 (18–70)	53 (26–68)	50 (18–70)	0.56
**Gender - n (%)**	Female	7 (4.7)	6 (40.0)	13 (43.3)	1.0
	Male	8 (5.3)	9 (60.0)	17 (56.7)	
**Underlying disease - n (%)**	Hodgkin lymphoma	5 (33.3)	4 (26.7)	9 (30.0)	0.55
	Non-Hodgkin lymphoma	1 (6.7)	3 (20.0)	4 (13.3)	
	Multiple myeloma	9 (60.0)	8 (53.3)	17 (56.7)	
**Conditioning regimen used - n (%)**	Bendamustine 180mg/m^2^ per day, 2 days; Melphalan 140mg/m^2^/ per day, 1 day.	5 (33.3)	6 (40.0)	11 (36.7)	0.78
	Etoposide 450 mg/m^2^ per day, 2 days; Melphalan 140 mg/m^2^per day, 1 day.	0 (0.0)	1 (6.7)	1 (3.3)	
	Melphalan 200 mg/m^2^	5 (33.3)	5 (33.3)	10 (33.3)	
	Melphalan 140 mg/m^2^	5 (33.3)	3 (20.0)	8 (26.7)	

The complete ASCT procedure, including cell mobilization, cell collection, administration of the conditioning regimen, stem cell infusion, and post-transplantation follow-up, was performed in an outpatient setting according to institutional guidelines.

## Study procedure

Patients were prospectively randomized one-by-one to either Arm 1 or Arm 2 when they underwent an ASCT. On Day +1 after transplantation, patients in Arm 1 received a single subcutaneous dose of 6 mg of the innovator pegfilgrastim whereas patients in Arm 2 received a single subcutaneous dose of 6 mg of a biosimilar pegfilgrastim. Complete blood cell counts were performed every 24 h until engraftment was confirmed. Patients were followed up daily and adverse events were cataloged in accordance with the Common Terminology Criteria for Adverse Events (CTCAE) version 5.0.

According to institutional guidelines, the historical Control Group had received a single daily subcutaneous dose of 5 µg/kg of filgrastim, starting on Day +1 post-transplantation and continuing until hematologic recovery was observed.

Neutrophil engraftment was defined as achieving an absolute neutrophil count of at least 0.5 × 10^9^/L for three consecutive days, whereas platelet engraftment was defined as a platelet count of at least 20 × 10^9^/L for three consecutive days without transfusion support.

FN neutropenia was defined as an absolute neutrophil count <0.5 × 10^9^/L with a single oral temperature reading of 38.3 °C or a sustained temperature of ≥38.0 °C over a one-hour period.

Transfusions administered to patients in both the prospective and retrospective groups were performed prophylactically, based on the standard operating procedure of the transplant unit, when the platelet count was less than 20 × 10^9^/L or hemoglobin was <7 g/dL. No patient required a therapeutic transfusion.

## Ethical considerations

This study received approval from the Institutional Ethics Committee (HE22–00006). All procedures were performed in accordance with the Declaration of Helsinki and Good Clinical Practice guidelines, with informed consent obtained in writing from each participant. The privacy rights of patients were always observed. This study was registered on the ClinicalTrials.gov platform under the identifier NCT05338047.

### Endpoints

The primary objective of this study was to compare times to neutrophil and platelet engraftment between two formulations of pegfilgrastim. Secondary aims included a comparative evaluation of safety and economic outcomes for these formulations versus daily filgrastim in patients undergoing outpatient ASCT. Parameters analyzed included transfusion and hospitalization rates, incidence of febrile neutropenia (FN), and total treatment costs.

### Statistical analysis

Data distributions were assessed using the Shapiro-Wilk test. Standard descriptive analyses were carried out to evaluate clinical and demographic characteristics. Between-group comparisons were made using the Mann-Whitney U and Chi-square tests. The times to neutrophil and platelet engraftment were calculated using the reverse Kaplan-Meier method and compared between treatment groups using the Log-Rank test. All statistical analyses were performed using IBM SPSS Statistics software (version 27; IBM Corp., Armonk, NY, USA). All tests were two-tailed, and a p-value of <0.05 was considered statistically significant. A post hoc power analysis was performed to assess the statistical power to detect a one-day difference in neutrophil engraftment between the innovator and biosimilar pegfilgrastim groups. Assuming a standard deviation of ±1.5 days, the study had an estimated power of 42 % with 15 patients per group and a two-sided α level of 0.05. These findings support the exploratory nature of this phase II equivalence study rather than one powered to detect superiority or non-inferiority

## Results

A total of 83 patients were included in the study; 30 patients were recruited prospectively and 53 were analyzed retrospectively (Control Group). Among the prospectively recruited patients, 15 patients received the innovator pegfilgrastim (Arm 1) and 15 patients received a biosimilar pegfilgrastim (Arm 2). The median age of all 83 patients was 50 years (range: 18–70 years) with no significant differences in age between groups (p-value = 0.56). Most of the patients in both Arms 1 and 2 were male (Arm 1: 53 % males; Arm 2: 60 % male). The underlying diseases and the conditioning regimens patients received are shown in Table 1.

### Engraftment and transfusion support

The median time to neutrophil engraftment was ten days in the innovator pegfilgrastim group and nine days in the biosimilar pegfilgrastim group (p-value = 0.14), whereas the median time to platelet engraftment was ten days in both groups (p-value = 0.81; Table 2). Regarding transfusion rates, only one patient in Arm 1 required a single red blood cell-packed transfusion (p-value = 0.31), whereas 13 (86 %) patients in each arm required platelet transfusions (p-value = 1.0). The number of platelet units transfused per patient ranged from 0–2 for patients in Arm 1 and from 0–3 for patients in Arm 2 (p-value = 0.21).

**Table 2:** Time to engraftment, blood transfusions, and adverse events by treatment arm.

Engraftment	Arm 1(Innovator pegfilgrastim)*n* = 15	Arm 2(Biosimilar pegfilgrastim)*n* = 15	Total*N* = 30	p-value
				
Neutrophil engraftment†	10 (9–11)	9 (7–13)	9 (7–13)	0.14
Platelet engraftment†	10 (8–13)	10 (8–18)	10 (8–18)	0.81
				
**Transfusions**				
Red cell packet transfused ‡	0	1	1	-
Platelet apheresis transfused ‡	1 (0–2)	2 (0–3)	1 (0–3)	0.21
				
**Complications**				
Hospitalization - n ( %)	1 (6.7)	1 (6.7)	2 (6.7)	1.0
Hospitalization days - median (range)	5 (3–10)	19 (3–19)	12 (3–19)	0.05
Neutropenic fever - n ( %)	1 (6.7)	1 (6.7)	2 (6.7)	1.0
Adverse events (grade I) - n ( %)	6 (40)	3 (20)	9 (30)	0.45
Adverse events (grade >II) - n ( %)	1 (6.6)	1 (6.6)	2 (6.6)	1.0
				
Cost in USD	541.60	492.90	-	0.001

† Median day post-transplant and range.

‡ Median and range of units transfused per patient until engraftment.

### Hospitalization and adverse events

Among all of the patients in the study, hospitalization was required for two patients (6.7 %) with a median hospital-stay of 12 days (range: 3–19 days); one patient from Arm 1 was admitted due to neutropenic fever and one from Arm 2 was admitted due to mucositis. The patient from Arm 1 had HL and died on Day +6 due to sepsis. The patient who was hospitalized for mucositis in Arm 2 had underlying chronic renal disease before ASCT that was associated with MM and required a 10-day stay in the intensive care unit due to metabolic and electrolytic disturbances not related to pegfilgrastim; this patient achieved engraftment on Day +13.

Both of the pegfilgrastim variants exhibited favorable safety profiles. Adverse events were observed in a total of 11 patients (36.7 %): seven (46.7 %) patients in Arm 1 and four (26.6 %) patients in Arm 2 (p-value = 0.45); the events were categorized as mild in most cases (Table 2). The most frequent adverse events that were probably related to the use of pegfilgrastim included Grade I/II nausea (13.4 %), myalgia (6.7 %), epigastric discomfort (6.7 %), weakness (3.3 %), and fever (6.7 %).

### Comparison between the pegfilgrastim and filgrastim groups

Fifty-three consecutive patients who received filgrastim were included as a Control Group and compared with those who received pegfilgrastim. The diagnoses of patients in the Control Group included HL (20.8 %), NHL (17 %), and MM (62.3 %). A comparison of demographic characteristics between these two groups is shown in Table 3. There were no statically significant differences between the groups for the analyzed variables, including age (p-value = 0.4), gender distribution (p-value = 0.6), and underlying disease (p-value = 0.6).

**Table 3:** Comparison of demographic characteristics between the study group (arm 1 + arm 2) and the Control Group.

Patient Characteristics	Pegfilgrastim(Arm 1 + Arm 2)	Filgrastim(Control Group)	p-value
	*n* = 30	*n* = 53	
Age in years - median (range)	50 (18–70)	56 (18–77)	0.4
Male gender - n ( %) Female gender - n ( %)	17 (56.7) 13 (43.3)	33 (62.2) 20 (37.8)	0.6
Diagnosis			0.6
Hodgkin lymphoma - n ( %)	9 (30.0)	11 (20.8)	
Non Hodgkin Lymphoma - n ( %)	4 (13.3)	9 (17.0)	
Multiple myeloma - n ( %)	17 (56.7)	33 (62.3)	

The median time to neutrophil engraftment was nine days in the pegfilgrastim group and 11 days in the filgrastim group (p-value = 0.0001), whereas the median times to platelet engraftment were ten days and 12 days (p-value = 0.0001), respectively (Table 4).

**Table 4:** Comparison of results between the study group (arm 1 + arm 2) and the Control Group.

Engraftment	Pegfilgrastim(Arm 1 + Arm 2)*n* = 30	Filgrastim(Control group)*n* = 53	p-value
			
Neutrophil engraftment†	+9 (7–13)	+11 (9–20)	0.0001
Platelet engraftment†	+10 (8–18)	+12 (9–20)	0.0001
			
**Transfusions**			
Red cell-packet transfused‡	1 (0–1)	1 (0–6)	0.063
Platelet-apheresis transfused‡	1 (0–3)	1 (0–4)	0.029
			
**Complications**			
Hospitalization - n ( %)	3 (10)	6 (11.3)	1.0
Hospitalization days - median (range)	3 (3–19)	3 (2–7)	0.71
Neutropenic fever - n ( %)	2 (6.7)	13 (24.5)	0.04
Identified infection - n ( %)	1 (3.3)	4 (7.5)	0.64
			
**Punctions and costs**			
Number of subcutaneous punctions - median (range)	1 (1)	11 (9–20)	0.0001
Cost in USD (range)§	517.20 (492.90–541.60)	425.9	0.001

† Median number of days post-transplant and range.

‡ Median number of units transfused per patient until engraftment and range.

§ Average cost between the innovator and biosimilar product.

With regard to transfusion requirements from the time of the administration of the conditioning regimen until hematological recovery, one patient (3 %) in the pegfilgrastim group and three patients (5.6 %) in the filgrastim group required at least one red cell-packed transfusion (p-value = 0.63). Regarding platelet transfusion, 26 patients (86 %) in the pegfilgrastim group and 39 patients (73 %) in the filgrastim group required at least one transfusion (p-value = 0.16). The number of red blood cell packs that were transfused per patient ranged from 0–1 in the pegfilgrastim group and from 0–6 in the filgrastim group (p-value = 0.063), whereas the number of apheresis platelet units that were transfused per patient ranged from 0–3 in the pegfilgrastim group and from 0–4 in the filgrastim group (p-value = 0.029).

The proportion of patients who required hospitalization was similar for the pegfilgrastim (10 %) and filgrastim (11.3 %) groups (p-value = 1.0). The median number of days that patients remained hospitalized was three days for both of these groups (p-value = 0.71); however, the incidence of neutropenic fever was statistically higher in the filgrastim group (24.5 % versus 6.7 %; p-value = 0.04; Table 4). A site of infection was identified in the five patients who presented with FN (two patients had cellulitis in the venipuncture region and three patients had diarrhea); however, blood cultures did not identify an isolated bacterial agent in any of these patients.

### Analysis of costs

The cost per patient treated was estimated in US dollars. Drug prices were obtained from the price list of the pharmacy in the hospital where the study was carried out using the prices from 2022. Regarding the cost of pegfilgrastim, the price of one dose of the innovator pegfilgrastim was higher than that of the biosimilar pegfilgrastim (USD 541.60 versus USD 492.90). The costs of the biosimilar pegfilgrastim and the biosimilar filgrastim differed significantly at USD 492.90 and USD 425.90 (p-value = 0.001), respectively; this cost included 11 administered doses of filgrastim (one vial daily from Day +1 to Day +11). In the cost analysis, only the cost of the medication was included, the cost of each application (nursing, transportation, etc.) was not included, even though pegfilgrastim is administered once only whereas filgrastim is administered once daily for 11 days. The discomfort caused to the patient by the different administration schemes of the two medications was also not considered in this analysis.

## Discussion

FN, a frequent complication in patients undergoing ASCT, increases morbidity, mortality, and hospitalization rates, thereby increasing transplantation costs. Neutropenic sepsis remains a significant life-threatening adverse event and cause of mortality in patients who receive chemotherapy from which patients undergoing transplantation are not exempt [6,7]. Various hematopoietic growth factors have been used, including filgrastim and pegfilgrastim with the aim of accelerating engraftment and reducing the number of days during which a patient is at risk of neutropenia [8]. This study analyzed the effectiveness of three different G-CSF formulations (innovator pegfilgrastim, biosimilar pegfilgrastim, and biosimilar filgrastim) based on the number of days to engraftment in patients undergoing outpatient ASCT.

A comparison between the innovator and biosimilar pegfilgrastim groups revealed no significant differences in demographic characteristics, underlying diseases, or conditioning regimens. (Table 1). Furthermore, there were no differences according to the type of G-CSF treatment received for the following variables: time to neutrophil engraftment (p-value = 0.14), time to platelet engraftment (p-value = 0.81), transfusion rate (p-value = 1.0), hospitalization rate (p-value = 1.0), FN frequency (p-value = 1.0), and frequency of adverse events (p-value = 0.45); this demonstrated the comparable effectiveness of a biosimilar pegfilgrastim and the innovator pegfilgrastim (Table 2). However, there was a difference between the costs of these medications (p-value = 0.001). Although this is not a phase III clinical trial and the number of patients is limited, in this study the effects of the two different pegfilgrastim products were comparable. In a previous study, Wong et al. compared the effectiveness of the innovator pegfilgrastim with a biosimilar pegfilgrastim to prevent FN in patients who had received chemotherapy for breast cancer; these authors observed no differences in the incidence of FN between treatments, but there was a significantly higher cost when the innovator medication was used [9].

On the other hand, differences in some variables were observed when comparing patients who received pegfilgrastim versus filgrastim including time to engraftment. Patients who received pegfilgrastim achieved neutrophil and platelet recovery two days faster than those who received filgrastim (p-value = 0.0001; Table 4). In the pegfilgrastim group, a greater number of patients required platelet transfusion support (86 % versus 73 %); however, the number of platelet units that were transfused was greater in the filgrastim group (p-value = 0.029) as many of the patients in the filgrastim group required more than one platelet transfusion.

The incidences of FN post-ASCT reported in the literature vary widely, ranging from 20–80 % [8,10]. Patients who receive an ASCT are considered to be at high risk for FN and for fatal complications with a combined mortality rate of up to 10 %; therefore, previous studies have recommended the use of filgrastim or pegfilgrastim for primary prophylaxis [11,12]. Some reports have shown that patients receiving pegfilgrastim have a lower incidence of FN and others have shown no differences in the incidence of FN when using filgrastim [8,13,14].

In the present study, the incidence of FN was significatively lower in patients who received pegfilgrastim (6.7 %) than in those who received filgrastim (24.5 %; p-value = 0.04). Similar results were observed in previous studies evaluating pegfilgrastim versus filgrastim for FN prophylaxis; these studies demonstrated that pegfilgrastim significantly reduced the risk of FN and its associated complications in chemotherapy patients [6,8].

The most common causes of hospitalization in patients receiving high doses of chemotherapy include mucositis and FN. The study center of this research is a FACT-accredited unit that has operated an outpatient autologous and allogeneic transplant program since 1998 [15]; the entire ASCT procedure is performed in the outpatient clinic. After chemotherapy administration and cell infusion, patients are discharged home and are then followed up in the outpatient clinic every day until engraftment. Patients are admitted to hospital if they develop FN or any other complication that requires in-hospital treatment; this does not include transfusions, which are performed in an outpatient setting. In the present study, we did not observe significant differences between the three groups of patients with respect to their hospitalization rate or length of hospital stay. The differences in the FN incidence and the hospitalization rate that were observed in the current study can be explained by the rapid response to outpatient antibiotic treatment that occurred in some patients such that they were not hospitalized.

The limitations of the present study are the small number of patients who were included, the lack of blinding, and the inclusion of a retrospective Control Group; however, it is important to highlight that there is little existing information on the outcomes of these patients in an outpatient setting.

## Conclusion

The two pegfilgrastim formulations demonstrated similar effects on time to engraftment and FN incidence. Compared to daily filgrastim, pegfilgrastim treatment resulted in faster engraftment, fewer platelet transfusions, and a lower incidence of FN. While pegfilgrastim was associated with a higher product cost, these clinical advantages may provide significant value in an outpatient setting.

## Funding sources

This research received no specific grant from any funding agency in the public, commercial or not-for-profit sectors. The study drug was funded by the institution where the research was conducted.

## Patient consent statement

Informed consent was obtained in writing from each participant

## Ethics approval statement

approval from the Institutional Ethics Committee (HE22–00006).

## Clinical trial registration

Identifier NCT05338047

## Author contributions

CHGA: Conceptualization, Investigation and writing original draft. FGS: Methodology and Investigation. DGA: Funding acquisition, writing – review & editing. JCJP: Writing review & editing. CMG: Project administration, Software. AGL: Formal analysis. PCP: Investigation and Validation. MJMM: Data curation and Formal analysis. VCER: Data curation and Formal analysis. OGCR: Investigation and Writing – review & editing.

## Data availability

The data that support the findings of this study are available from the corresponding author upon reasonable request.

## Conflicts of interest

The authors declare that they have no conflict of interests.
